# Optimizing hybrid assembly of next-generation sequence data from *Enterococcus faecium*: a microbe with highly divergent genome

**DOI:** 10.1186/1752-0509-6-S3-S21

**Published:** 2012-12-17

**Authors:** Yajun Wang, Yao Yu, Bohu Pan, Pei Hao, Yixue Li, Zhifeng Shao, Xiaogang Xu, Xuan Li

**Affiliations:** 1 Shanghai Center for Systems Biomedicine, Shanghai Jiaotong University, Shanghai 200240, China; 2 Key Laboratory of Synthetic Biology, Institute of Plant Physiology and Ecology, Shanghai Institutes for Biological Sciences, Chinese Academy of Sciences, Shanghai 200032, China; 3 Institute of Antibiotics, Huashan Hospital of Fudan University, Shanghai 200040, China; 4 Institut Pasteur of Shanghai, Chinese Academy of Science, Shanghai 200025, China

## Abstract

**Background:**

Sequencing of bacterial genomes became an essential approach to study pathogen virulence and the phylogenetic relationship among close related strains. Bacterium *Enterococcus faecium *emerged as an important nosocomial pathogen that were often associated with resistance to common antibiotics in hospitals. With highly divergent gene contents, it presented a challenge to the next generation sequencing (NGS) technologies featuring high-throughput and shorter read-length. This study was designed to investigate the properties and systematic biases of NGS technologies and evaluate critical parameters influencing the outcomes of hybrid assemblies using combinations of NGS data.

**Results:**

A hospital strain of *E. faecium *was sequenced using three different NGS platforms: 454 GS-FLX, Illumina GAIIx, and ABI SOLiD4.0, to approximately 28-, 500-, and 400-fold coverage depth. We built a pipeline that merged contigs from each NGS data into hybrid assemblies. The results revealed that each single NGS assembly had a ceiling in continuity that could not be overcome by simply increasing data coverage depth. Each NGS technology displayed some intrinsic properties, i.e. base calling error, systematic bias, etc. The gaps and low coverage regions of each NGS assembly were associated with lower GC contents. In order to optimize the hybrid assembly approach, we tested with varying amount and different combination of NGS data, and obtained optimal conditions for assembly continuity. We also, for the first time, showed that SOLiD data could help make much improved assemblies of *E. faecium *genome using the hybrid approach when combined with other type of NGS data.

**Conclusions:**

The current study addressed the difficult issue of how to most effectively construct a complete microbial genome using today's state of the art sequencing technologies. We characterized the sequence data and genome assembly from each NGS technologies, tested conditions for hybrid assembly with combinations of NGS data, and obtained optimized parameters for achieving most cost-efficiency assembly. Our study helped form some guidelines to direct genomic work on other microorganisms, thus have important practical implications.

## Background

Sequencing of bacterial genomes is an essential approach to understand the virulent mechanisms of pathogens and the evolutionary relationship among close related pathogenic strains. Bacterial isolates of the same species often display surprisingly highly divergent gene contents from vastly different ecological environments. Such genome divergence, which is the result of harsh selection and frequent horizontal gene transfer, presents a unique challenge to the modern sequencing technologies featuring high-throughput and short read length, and limits our ability to re-sequence and construct a genome draft of bacterial variant by taking advantage of a "genome reference".

The case of bacterium *Enterococcus faecium *presents a unique example of such challenge. *E. faecium *emerged as an important nosocomial pathogen from hospital environments and were often associated with the resistance to many common antibiotics. It was a major cause of infections in hospitalized immuno-deficient patients [1]. *E. faecium *is a Gram-positive bacterium with a genome size of roughly 3 Mb. The first draft genome of *E. faecium *strain TX0016 was assembled in 2000. Since then, more than 25 partial genomes were published in succession [1]. The lack of a complete genome sequence of *E. faecium *might be due to factors including genome plasticity of *E. faecium *that harbors large insertions and deletions [2], and abundant repetitive sequences that hinder the assembly of a complete genome from sequence reads [2,3]. A genome sequence of a vancomycin-resistant *E. faecium *strain (Aus0004) was completed more recently [4], revealing large segments of repetitive DNA and insertion sequence elements in its genome.

The development of new generation sequencing technologies, e.g. 454 GS-FLX, Illumina Genome Analyzer, ABI SOLiD, PacificBio SMRT, etc, made it possible to sequence a bacterial genome with considerably less cost [5,6]. While the new generation sequencing technologies are attractive for sequencing and constructing bacterial genomes, there are some major factors that seriously impact the performance of such approach. Among them, the short sequence reads, high base calling error rates, and systematic bias of the next generation sequencers were often cited as drawbacks that made de novo genome assembly difficult, incomplete, and/or erroneous [5,7]. To address these issues and obtain high quality genomes, one common approach taken by researchers was to increase the coverage depth of sequencing reads [8]. Genome drafts of *Helicobacter acinonychis *[8] and panda [9] were constructed based on such method. However, such approach not only reduced the cost-effect benefit of nextgen sequencing technologies, but also failed to reduce the systematic bias of the sequencing platform. Another approach attempted by some scientists was to combine sequence data from different technologies, thus in theory they could correct sequence error/bias and improve the quality of draft genome. This so called "hybrid" approach was adapted by the combinations of 454 and Illumina data [10,11], and 454, Illumina and Sanger data [12]. While the hybrid approach achieved a higher efficiency in genome assembly, the most cost-effective SOLiD data was somewhat excluded in all these studies. Whether SOLiD data could significantly contribute to the hybrid assembly method remained an open question. In addition, there were many variables that influenced the outcome of a hybrid assembly. To investigate how these parameters affect the quality of genome assemblies and how to achieve most cost-efficiency in designing a "hybrid" project were the main goals of the present study.

In the current study, we were presented with an opportunity to attempt the hybrid approach in assembling a variant strain of nosocomial pathogen *E. faecium*, a medically important microbe. As mentioned above, the high genome divergence of *E. faecium *had prevented the completion of a genome draft although at least twenty-eight different variant strains had been sequenced, some to very high coverage depth. Under such scenario, we first sequenced the *E. faecium *variant isolated from a hospitalized patient using three different next-generation sequencing (NGS) technologies: 454 GS-FLX (454), Illumina GAIIx (GAIIx) and ABI SOLiD4.0 (SOLiD). We built a new analysis pipeline: 1) to perform primary assemblies with each single NGS data, by which established a baseline from each single NGS data to compare results and evaluate parameters for hybrid assemblies; 2) to perform secondary assemblies with the combinations of two or three single NGS data. With these design we characterized some systematic error and bias for each NGS platform, and were able to optimize parameters for performing hybrid assembly. Our results revealed that hybrid assembly method greatly improved efficiency in comparison with single NGS technology, which could not be achieved by simply increasing the coverage depth of a single NGS platform alone. We also assessed a number of parameters that would help guide the design and preparation of hybrid assembly studies of bacterial genomes.

## Methods

### DNA preparation and sequencing

The origin of *Enterococcus faecium *strain was isolated from a patient's peritoneal drainage fluids in HuaShan hospital. The genomic DNA of this bacterium was extracted and prepared for sequencing with three sequencers. For the Roche GS-FLX platform, the SR library was constructed and sequenced with methods described by Margulies and his co-workers [6]. The preparing the PE library and sequencing on the Illumina GAIIx sequencer were performed according to the standard Illumina protocols (Illumina, San Diego, CA, USA). For sequencing on the SOLiD 4 system, the 50-base SR library preparing, sequencing and base calling were performed according the manufacturer's recommendations (Applied Biosystems, Carlsbad, California, USA).

### Primary assembly

**Figure 1:**
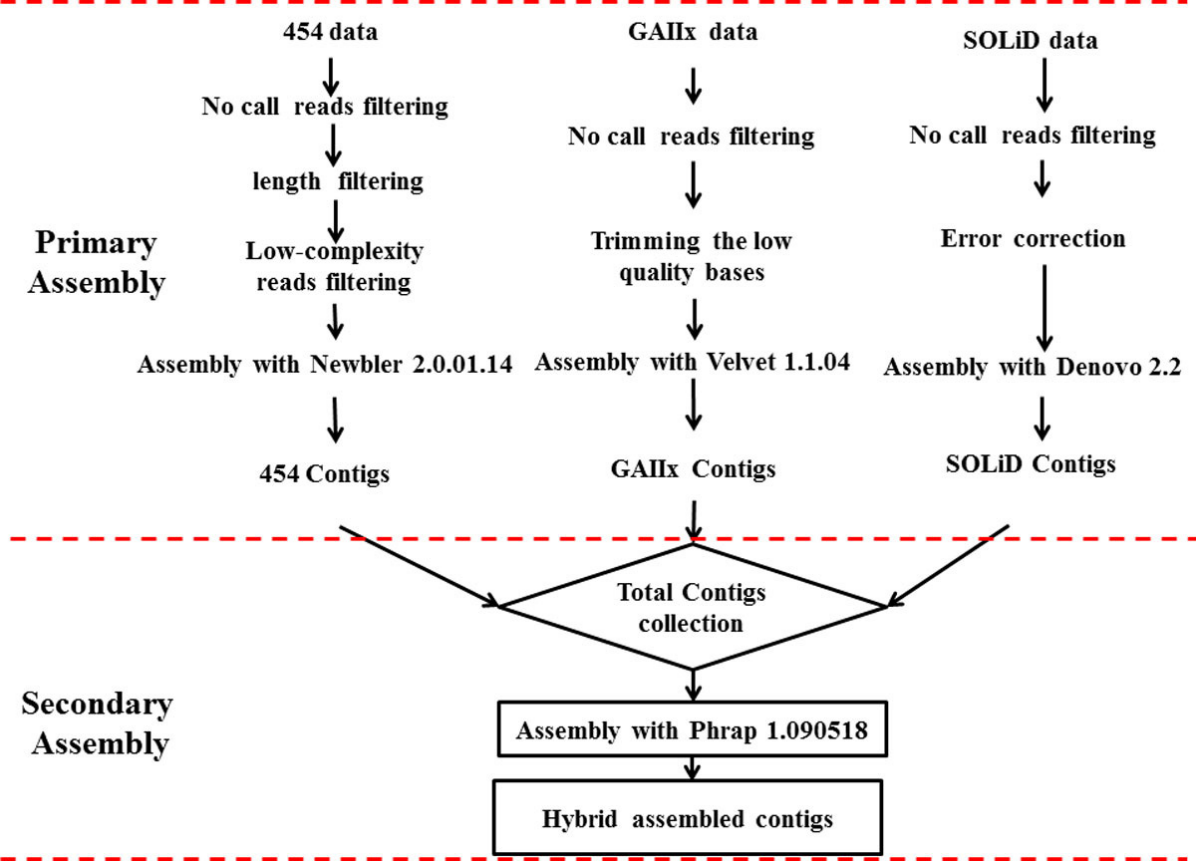
**The pipeline for hybrid assembly**. The pipeline for hybrid assembly consisted of primary and secondary assembly steps. In primary assembly, the 454, GAIIx, or SOLiD reads were assembled into contigs with Newbler2.0.01.14, Velvet1.1.04, or Denove2.2, respectively. In secondary assembly, contigs from primary assembly step were merged into consensus genome draft with Phrap(Ver1.090518). The consensus was then used as a reference of *E.faecium *for further analysis.

Before assembly, the raw data were pre-processed to filter the low quality reads (Figure 1). For 454 raw data, we discarded reads that contained uncalled bases (no-calls), and discarded the reads whose lengths were less than 50 bp or longer than 1000 bp. The reads containing low complexity sequences were filtered out using the method of DUST with threshold of 7, and the software of PRINSEQ was used to perform the work [13]. After pre-processing, the 454 dataset were assembled with assembler of Newbler2.0.01.14; and the default parameters were applied.

For Illumina raw data, the reads containing uncalled base positions were removed firstly. Then, we trimmed the low quality bases with the PERL script of fastq_qualitytrim_window.pl from http://xyala.cap.ed.ac.uk/Gene_Pool/scripts.tar.gz. The parameters of quality threshold and window size were set as 20 and 2 respectively. After pre-processing, the high quality Solexa dataset was assembled with velvet1.1.04 [14], the optimized k-mer parameter (hash length) of 31 was used to perform assembly after testing a series of k-mers (from 21 to 71 with interval of five).

For the SOLiD raw data, the reads including the uncalled base was removed, and then the remaining reads were corrected with the program of SAET. We assembled the SOLiD data with Denovo2.2 pipeline http://www.appliedbiosystems.com.cn/ . The optimized k-mer parameter of 25 was applied for the assembly.

### Secondary assembly

For each platform, the preassembled contigs whose length was less than 100 were discarded. The remaining contigs were aligned to the plasmids library in NCBI ftp://ftp.ncbi.nlm.nih.gov/genomes/Plasmids/ with blastn [15]. The contigs, in which 60% bases could be aligned to the plasmids with identity over 90%, were considered as plasmids sequences, and these contigs were abandoned before performing secondary assembly. We then collected the remaining contigs from the three platforms and performed the secondary assembly with Phrap1.090518 [16] (Figure 1). To make the overlap to be more specific, we set the parameter of minimum matching length as 50, -repeat_stringency as 0.95 and default_qual 30. In addition, the secondarily assembled contigs, which were extended with only GAIIx data or SOLiD data, were removed.

### Assembled contigs analysis

It is difficult to select a suitable reference of *Enterococcus faecium*, because of the high variance among stains, therefore we took the hybrid contigs from three platforms as reference genome, and we named this group of contigs as HEf-3 (GenBank: AJTW00000000). For each platform, we aligned preassembled contigs to HEf-3 respectively. The software Mummer3 [17] was used to perform the pairwise alignment and the default parameters were set. After the alignment, the regions in the reference not covered by the contigs from each platform were considered as gaps in the corresponding platform. Additionally, 28 achieved drafts *Enterococcus faecium *genomes in NCBI http://www.ncbi.nlm.nih.gov/genome/ were aligned to HEf-3 to evaluate our assembled contigs with Mummer3. (GenBank:NZ_ABQA00000000, GenBank:NZ_ABQI00000000, GenBank:NZ_ABQJ00000000, GenBank:NZ_ABRY00000000, GenBank:NZ_ABSC00000000, GenBank:NZ_ABSW00000000, GenBank:NZ_ACAS00000000, GenBank:NZ_ACAX00000000, GenBank:NZ_ACAY00000000, GenBank:NZ_ACAZ00000000, GenBank:NZ_ACBA00000000, GenBank:NZ_ACBB00000000, GenBank:NZ_ACBC00000000, GenBank:NZ_ACBD00000000, GenBank:NZ_ACHL00000000, GenBank:NZ_ACIY00000000, GenBank:NZ_ACJQ00000000, GenBank:NZ_ACOB00000000, GenBank:NZ_ACOS00000000, GenBank:NZ_ACZZ00000000, GenBank:NZ_ADMM00000000, GenBank:NZ_AEBC00000000, GenBank:NZ_AEBU00000000, GenBank:NZ_AECH00000000, GenBank:NZ_AECI00000000, GenBank:NZ_AECJ00000000, GenBank:NZ_AAAK00000000, GenBank:NZ_AEBG00000000).

### Sequencing Bias analysis from three NGS platforms

The coverage depth along HEf-3 was evaluated by mapping the reads from each platform to HEf-3. For 454, Illumina and SOLiD reads, we used Newbler2.0.01.14, BWA0.5.9-r16 [18], and Bioscope1.3 http://www.appliedbiosystems.com.cn/ to perform the alignment with default parameters, respectively. Subsequently, for each base on the reference, we calculated the number of reads that covered it. For each platform, the GC content in the regions of bottom and top 5% coverage depth was analysed.

After mapping the reads to HEf-3, we analysed the substitution errors from each platform. The bases in the mapped reads, which were not consistent with the bases in the HEf-3, were considered as substitution errors. 12 types of substitution errors from three platforms were calculated and compared.

The k-mer depth, which defined as the number of times that a k-mer appears in the sequencing data, was used to identify the repetitive sequence of genome [19]. A higher k-mer depth indicated this k-mer is more likely to appear in repeat regions. For k-mer bias comparison, the k-mers, whose depth ranked on the top10000 of total k-mers, were extracted with JAVA script firstly. Hence, for each NGS platform, the depth of these k-mers was normalized by dividing the depth of total k-mers. Finally, the normalized k-mers depth was compared among the three NGS data with density diagram.

### Optimizing parameters for hybrid assembly

To optimize the hybrid assembly, we investigated how the assembled contigs from one platform were influenced by different amount of data from another platform. Based on the pipeline in Figure 1, all the data from each platform were firstly preassembled, for example 454 data, then we randomly sample different amount of data from another platform (GAIIx data or SOLiD) and preassembly these data. For each subgroup sampling, we repeated 5 times from our sequencing data pool. Finally the secondary assembly was performed with these two groups of preassembled contigs using Phrap. The assembly parameters were same as that used above. Similarly, the performance of assembled Illunima contigs influenced by subgroup data from 454 and SOLiD platform, and the performance of assembled SOLiD contigs influenced by subgroup data from 454 and GAIIx platform, were also studied.

## Results

### Generating and processing sequence data from NGS platforms

In order to investigate the properties of sequencing data from each NGS platform and combine them to achieve the best genome assembly, we sequenced the nosocomial pathogen *Enterococcus faecium *with the three popular NGS platforms: Roche 454, Illumina GAIIx, and ABI SOLiD 4. We refer them as 454, GAIIx, and SOLiD hereafter. This strain of bacterium was originally isolated from a patient's peritoneal drainage fluids. It was reported that *E.faecium *showed a high degree of genomic variations among many closely related strains [1], which presented a mounting challenge to characterize the complete genome of a variable strain.

The sequencing data were summarized in Table 1. For GAIIx, we sequenced the *E. faecium *strain with 300 bp paired-end library and read length of 70 bp. We obtained ~500-fold coverage of reads. For 454, we generated approximately 28-fold sequence coverage using SR library with an average read length of 212 bp. For SOLiD, we obtained approximately 400-fold sequence coverage using SR library with a read length of 50 bp. The sequence reads from each NGS platform were processed and passed quality filters separately based on standard protocols as described in Methods. On average, 76.9%, 99.84%, and 99.08% of data were retained from GAIIx, 454, and SOLiD, respectively (Table 1), and were subjected to subsequent studies.

**Table 1:** Sequence data obtained from three NGS platforms for the *E.faecium *strain.

NGS Platform	Number of Reads	Read Type	Average Read Length (bp)	Total Base (bp)	Estimated Coverage Depth
**454**	424,370	SR	212	89,966,440	~28 ×
**GAIIx**	21,525,592	PE	70	1,506,791,440	~500 ×
**SOLiD**	26,568,626	SR	50	1,328,431,300	~400 ×

### Assemblies with single-NGS data sets

To evaluate the assemblies from each single-NGS platform and generate a baseline for comparative analysis, we first constructed genome drafts for the *E. faecium *strain from each single-NGS data set. The default assembling programs developed or recommended by the sequencer manufacturers were employed: Newbler 2.0.01.14 for 454 and Denovo 2.2 for SOLiD data sets. In the case of GAIIx data set, we chose Velvet1.1.04 from among a number of assemblers recommended by Illumina. The assembling process for each single-NGS data set was illustrated in the primary assembly step in Figure 1. The performance of each single-NGS assembly was measured with genome draft size, number of contigs, and the N50 size, which are summarized in Table 2.

**Table 2:** Genome drafts of the *E.faecium *strain from primary and secondary assemblies.

NGS Data		Genome Draft Size (bp)	Number of Contigs	N50 (bp)	Max Contig (bp)
**Primary**	**454**	3,027,996	352	21,989	92,031
	**GAIIx**	2,947,516	1,013	9,804	45,465
	**SOLiD**	2,976,193	3,385	1,389	7,396
**Secondary**	**454+GAIIx+SOLiD**	3,103,094	204	34,849	114,611
	**454+GAIIx**	2,971,758	209	34,597	114,610
	**454+SOLiD**	3,018,371	257	23,443	97,560
	**GAIIx+SOLiD**	2,923,382	465	13,773	66,062

### Assembly with 454 data set

**Figure 2:**
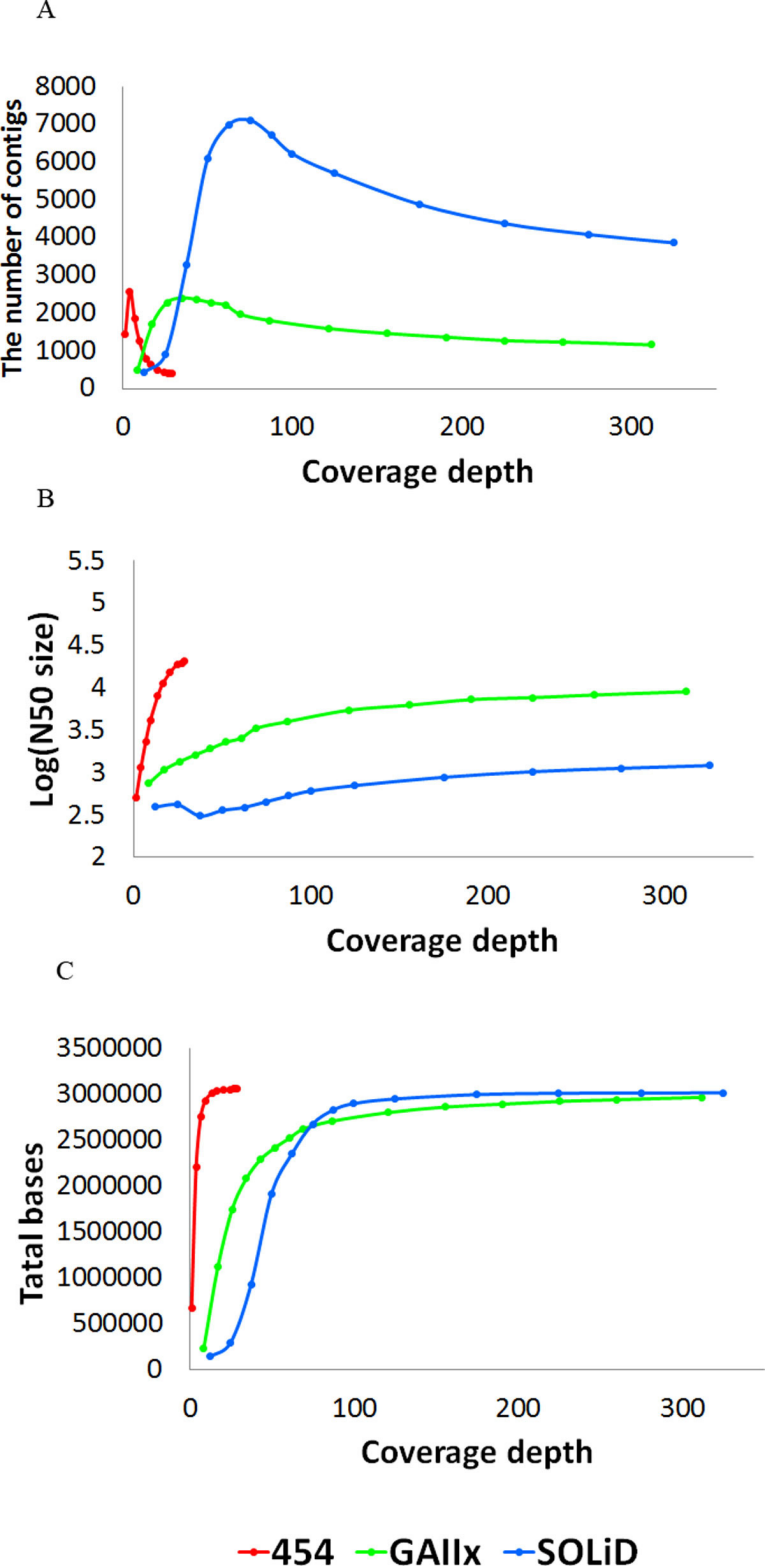
**The performance of assembly as increasing coverage depths**. The performance of assembled contigs (A), N50 size (B) and total bases (C) for the three NGS technologies at increasing depths in simulation. The red line, green line and blue line show the performance of Roche 454, Illumina GAIIx, and ABI SOLiD platform, respectively. Reads from each platform were simulated by random subsampling. Summary of performance of each platform, 25×, 240× and 300× sequencing data generating from 454, GAIIx and SOLiD platforms was sufficient for de novo assembly.

Using the 454 dataset, a genome draft of 3.028 MB was constructed with an N50 size of 21,989 bp and 352 contigs (Table 2) after discarding the plasmids sequence. We then analysed the effect of coverage depth on assembly result by varying the amount of sequence data used in assembly. As shown in Figure 2, while the number of contigs increased first and reduced to ~400 at about 20-fold coverage, the draft genome size reached a flat line at 10-fold coverage depth and remained stable above 10. However, the N50 size of genome draft continued to increase until levelled out at 25-fold coverage.

### Assembly with GAIIx data set

Using the GAIIx dataset, a genome draft of 2.948 MB was constructed, with N50 size of 9,804 bp and 1013 contigs (Table 2). We then randomly sampled GAIIx reads and performed assembly with variable coverage depth. As shown in Figure 2, the draft genome reached a flat line at 200-fold coverage depth, while the number of contigs increased to ~2500 before fell back to ~1100 at about 180-fold coverage depth. The N50 size of genome draft continued to increase until levelled out at 240-fold coverage depth. Further increasing the sequence reads beyond 240 seems to affect very little on the assembly results. The higher number of contigs and smaller N50 size are the result of GAIIx short read length compared to 454 sequence data.

### Assembly with SOLiD data set

Using the SOLiD dataset, a genome draft of 2.98 MB was constructed, with an N50 size of 1,389 bp and number of contigs is 3,385 (Table 2). We also randomly sampled SOLiD reads to vary the coverage depth for assembly with Denovo2.2. As shown in Figure 2, the draft genome reached a flat line at 100-fold coverage depth, while the number of contigs increased to 7500 before fell back to ~4000 at about 220-fold coverage depth. The N50 size of genome draft continued to increase until levelled out at 300x coverage depth. Further increasing the sequence reads beyond 300 had very little effect on assembly outcome. Similar, the higher number of contigs and smaller N50 size are the result of short read length from SOLiD. In comparison with assembly results from 454 or GAIIx data, SOLiD data produced the worst draft in term of sequence continuity.

### Hybrid assembly with all NGS data

The genome drafts produced from single-NGS dataset indicated that each NGS platform had some intrinsic "defect" that limited the quality of genomic draft. Such inherent limitation with each NGS data can't be overcome by simply increasing data coverage depth as illustrated by our results (Figure 2). Furthermore, each NGS platform displayed different efficiency in forming assemblies; 454 was the foremost in creating a higher degree of contiguity of genome assembly over the GAIIx and SOLiD data.

In order to remedy the single-NGS limitation, we attempted with hybrid assembly approach by merging each single-NGS assemblies through a secondary assembly step. The process for hybrid assembly is outlined in Figure 1. The contigs constructed from primary assembly step were merged using Phrap (ver 1.090518). The hybrid assembly with all three NGS data, named HEf-3 (Table 2), formed a consensus that had a total size of 3,103,094. The consensus genome draft had its number of contigs reduced to 204, and N50 size increased to 34,849 bp (Table 2).

Hybrid assembly was also attempted by combining two of the three single-NGS data. As shown in Table 2, the combinations of two single-NGS assemblies also improved secondary assembly remarkably over primary assemblies.

### Evaluating results of hybrid assemblies

**Figure 3:**
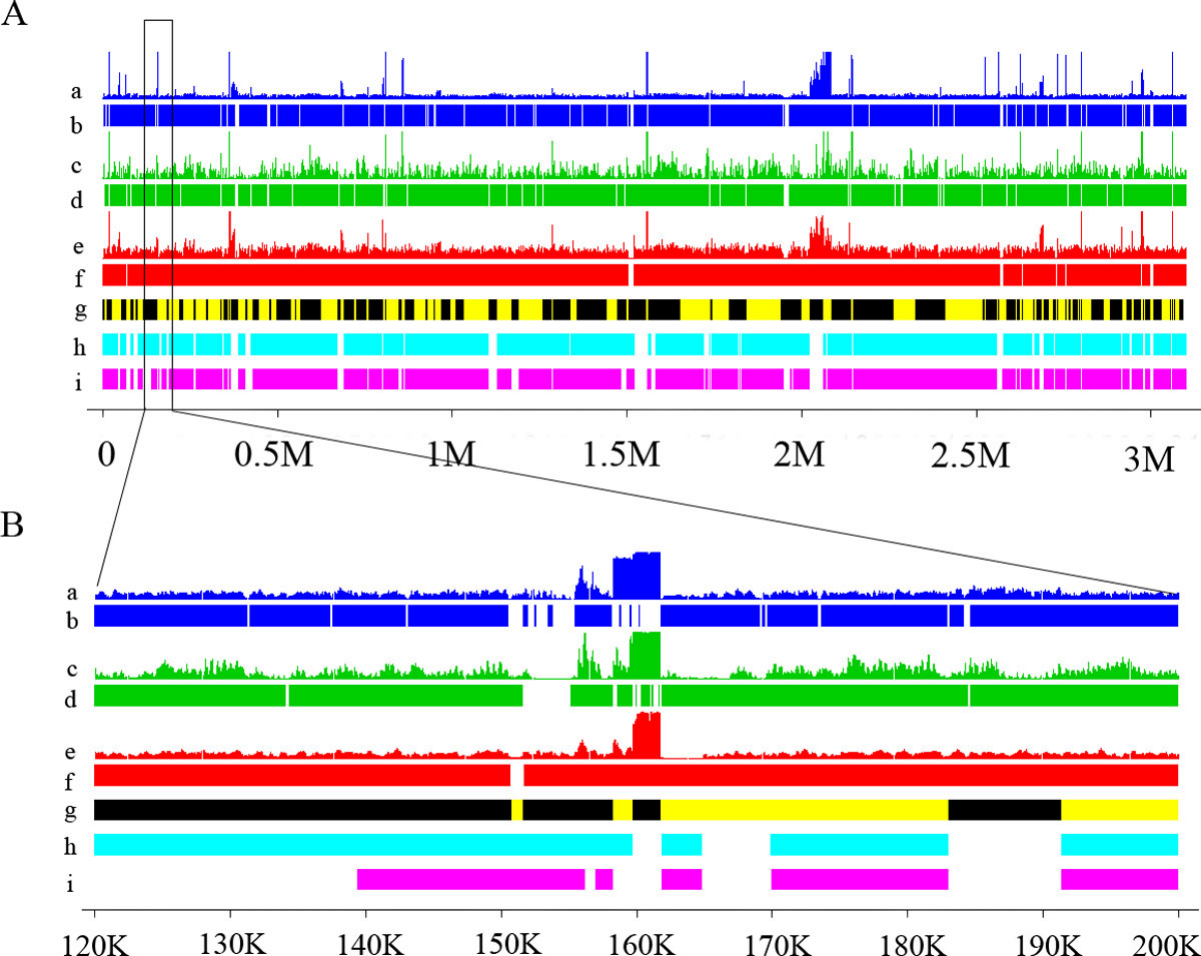
**Comparison of genome assemblies**. (A) And (B) Comparison of genome assemblies, (a) Distribution of SOLiD reads along consensus genome assembly; (b) Genome assembly from SOLiD data. (c) Distribution of Solexa reads along the consensus genome assembly; (d) Genome assembly from Illumina data; (e) Distribution of 454 reads along the consensus genome assembly; (f) Genome assembly from 454 data; (g) Consensus genome assembly from hybrid assembly, The adjacent contigs are distinguished by yellow and black colors; (h) Genomes assembly of an *E. faecium *(GenBank:NZ_ABSW00000000); (i) Genomes assembly of an *E. faecium *(GenBank:NZ_ACAX00000000); The gap regions was displayed in white color (B) Magnification of regions from 120 K to 200 K.

To evaluate the results from hybrid genome draft, we compared the HEf-3 assembly with those from single-NGS data and with the genome drafts of 28 other *E. faecium *strains deposited at NCBI. As shown in Figure 3, the HEf-3 genome draft was aligned with 454, GAIIx, SOLiD drafts, and with the two most close genome sequences of *E. faecium *strains U0317 and 1,231,502 (GenBank: NZ_ABSW00000000, GenBank: NZ_ACAX00000000). Each NGS platform displayed a distinct pattern of coverage depth along its genome sequence. Particularly the gaps resulted from low read coverage were staged differently in each single-NGS assembly, which made it possible to fill in the gaps by merging the contigs. In HEf-3, the number of gaps was reduced by 42% compared to the best single-NGS assembly from 454.

When compared with 28 other *E. faecium *strains, two *E. faecium *draft genomes, U0317 (GenBank: NZ_ABSW00000000), and 1,231,502 (GenBank: NZ_ACAX00000000), which are 2,893,029 bp and 2,926,114 bp in size, respectively, showed the highest similarities to HEf-3. They had 93.8% and 90.6% overall identities to HEf-3 in the aligned regions (Figure 3). The N50 sizes of the two genome drafts were 31,583 bp and 28,295 bp respectively, smaller than that of HEf-3 (34,849 bp). The HEf-3 draft, generated with the hybrid assembly approach improved the genome draft of *E. faecium*, resulting in a quality assembly better over these two reported *E. faecium *genome above. The consensus genome from HEf-3 was then used as a reference for further analysis.

### Analysis of sequencing biases from three NGS platforms

We demonstrated the hybrid assembly approach generated a superior genome draft than those from single NGS data. The data also clearly suggested that each NGS platform had some systematic but distinct biases towards each base of *E. faecium *genome.

#### Bias for coverage depth

It was observed that the reads aligned to genome assembly were not distributed uniformly among different platforms. The 454 reads were more uniformly mapped than any of the other two (Figure 3). To quantify the extent of biases in each NGS, we first measured the variations of coverage depth. The standard deviation of coverage depth for 454, GAIIx, and SOLiD was 25.5, 400.7 and 426.4, respectively. The results indicated SOLiD and GAIIx had a higher coverage variation than 454. We then computed the correlation coefficients (r) of per-base coverage depth among 454, GAIIx, and SOLiD. The coefficients (r) between 454 and GAIIx, 454 and SOLiD, and GAIIx and SOLiD on the same sample were 0.698, 0.690, and 0.747, respectively. These numbers indicated a comparatively stronger correlation between GAIIx and SOLiD than correlation between 454 and GAIIx, and 454 and SOLiD.

#### Bias for GC contents

The HEf-3 assembly had an average GC content of 37.6%. To test whether uneven distribution of GC base caused the variation of coverage depth on each NGS reads, we measured the GC contents in high or low coverage regions, and in gap regions in 454, GAIIx, and SOLiD assemblies separately. Among the top 5% covered bases, the GC contents were 37.7%, 43.3%, 36.4% for 454, GAIIx, and SOLiD, respectively. Among the bottom 5% covered bases the GC contents were 31.5%, 29.9%, 27.7%. And lastly, in the gapped regions the GC contents were 36.6%, 33.1%, 33.9% (Table 3).

**Table 3:** GC content in different regions of *E.faecium *genome

Platform	Avg GC% content	GC% in regions of bottom 5% coverage depth	GC% in regions of top 5% coverage depth
**Solexa**	37.8	29.9	43.3
**454**	37.7	31.5	37.7
**SOLiD**	38.3	27.7	36.4

**Figure 4:**
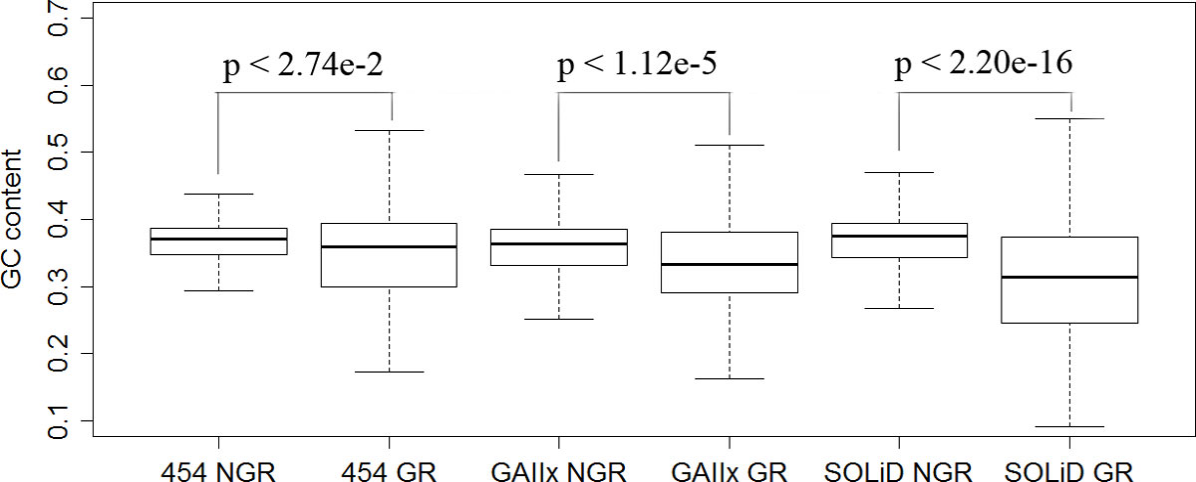
**GC contents comparison between gap regions and non-gap regions**. The gap regions (GR) have a comparative lower GC content compared with non-gap (NGR) regions. Contigs from SOLiD platform are most sensitive to the GC content. Here the gap region means the some continuous bases existing in the reference were not covered by the contigs from any platform when they were aligned to the reference.

The data indicated that the GC contents had significant influence on coverage depth, with the p-values (top 5% vs bottom 5%; Welch Two Sample t-test) being 2.2e-16, 2.2e-16, 2.2e-16 for 454, GAIIx, and SOLiD, respectively. The data also suggested that the GC contents had a great impact on coverage depth to all of the three kinds of reads. Although the low coverage regions of 454 were greatly influenced by the GC contents, its high coverage regions weren't significantly impacted by the GC contents. For each NGS platform, the GC content was compared between gapped and non-gapped regions (Figure 4). The p-values for 454, GAIIx, and SOLiD are 2.74e-2, 1.12e-5 and 2.20e-16, respectively. The statistics indicated that gaps from SOLiD data were most likely influenced by GC contents. Based on the analysis above, we found the GC contents greatly influenced the coverage depth and continuity of genome, especially assembled with GAIIx and SOLiD data.

#### Bias for k-mer diversity

**Figure 5:**
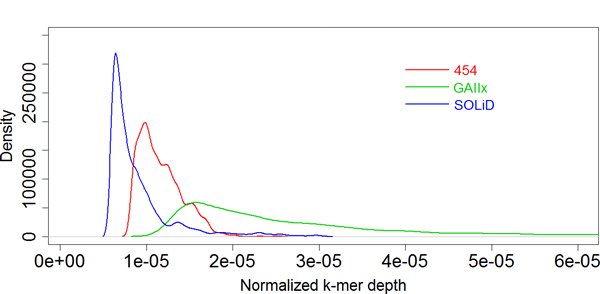
**Distribution of k-mer depth from three platforms**. The k-mers, whose depth ranked on the top 10000 of total k-mers, were used for comparison. The distribution representing 454, GAIIx and SOLiD k-mer depth is marked with the colour of red, green and blue, respectively.

The genomes were often interrupted in the repetitive regions during de novo assembly [7]. These repetitive regions could be detected with the k-mers, and a higher k-mer depth (see Methods for k-mer depth definition) indicated a higher rate of appearing in repeat regions [19]. In our studies, the k-mers with a comparatively high depth (Ranked on the top 10000 of total k-mers) was selected and compared among three NGS data (Figure 5). For these k-mers, the distribution of k-mer depth demonstrated that GAIIx have a highest k-mer depth, 454 had a moderate depth and SOLiD have a lowest k-mer depth (Figure 5). A higher k-mer depth from GAIIx than 454 might be influenced by high GC content (40% for GAIIx and 40.2% for 454). But lowest k-mer depth from SOLiD data was mainly affected by the errors. Because the SOLiD reads were encoded with color-space, an error in a read would make the rest of that read (from error position to the 3'end) generate a group of completely different k-mers, which forced the k-mers to be more diverse, thus the depth of every k-mer would decrease.

#### Bias for substitution error

**Figure 6:**
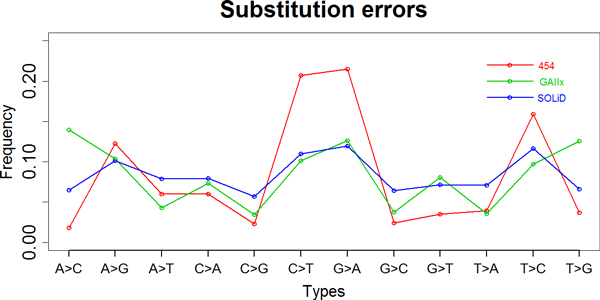
**Substitution errors from three NGS data**. Ratio of substitution errors in all kinds of substitution errors generated from three platforms. The errors from 454, GAIIx and SOLiD platforms are marked with the colour of red, green and blue, respectively.

The single-base substitutions of three NGS platforms were evaluated using HEf-3 consensus draft as a reference. We examined bases from sequencing reads of each NGS platform. There are 12 different types of substitution error. Overall, there were a lower rate of C to G and G to C substitution error, and a higher rate of C to T and G to A substitution error from all the three platforms (Figure 6). Each NGS platform showed a different error rate for some types of base substitution error. For example, base "A" in Solexa reads was more likely to change to C (14.0% of all substitution errors), Dohm also demonstrated a higher rate of A to C error of Solexa data [20]. However, in 454 and SOLiD reads it was merely 1.8% and 6.5%, respectively.

### Optimizing parameters for hybrid assembly

**Figure 7:**
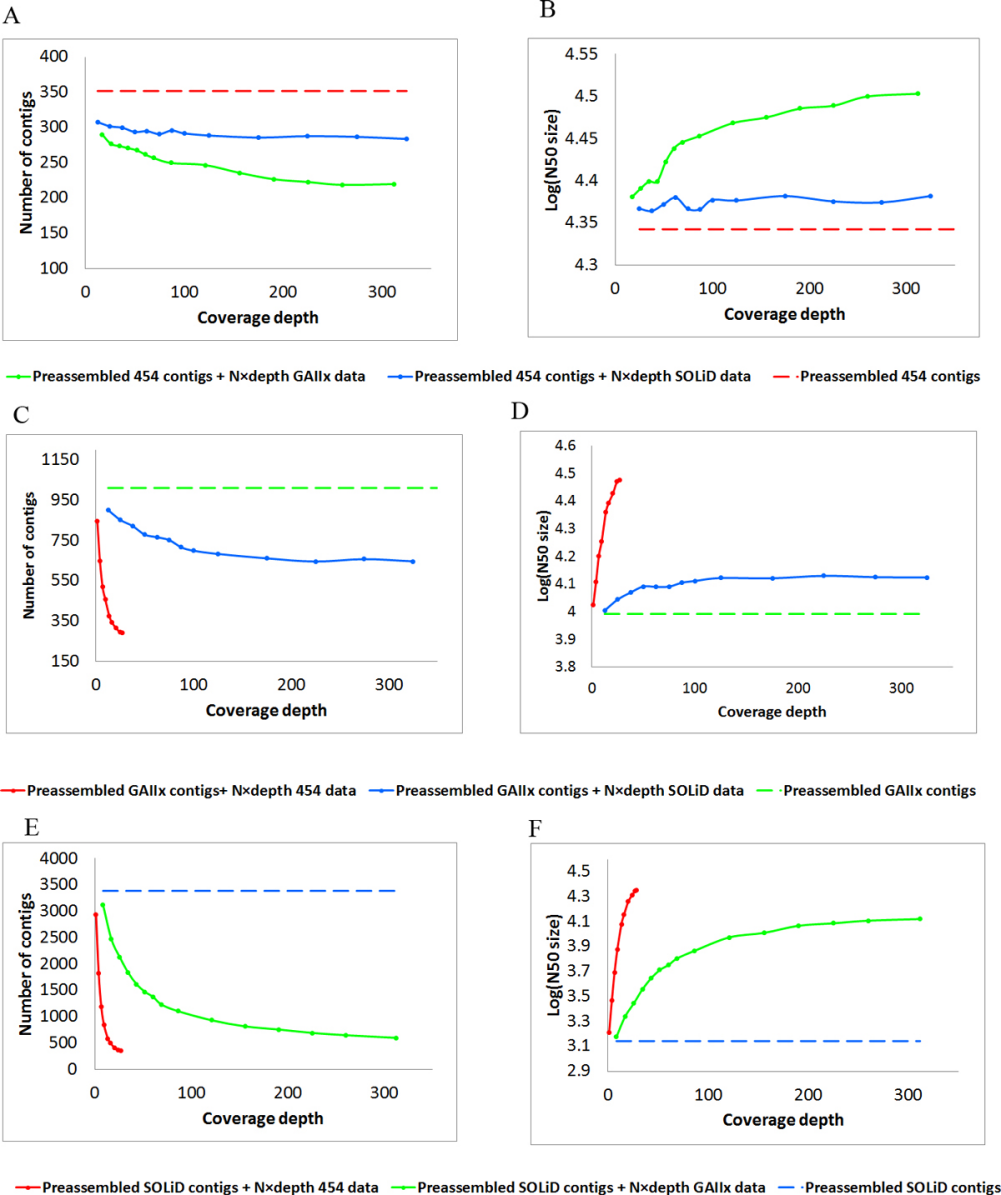
**Hybrid assembly influenced by different NGS reads at increasing depths**. The improvement of N50 size of 454 contigs, GAIIx contigs, SOLiD contigs by adding different depth of other data were shown in Figure B, D and F. And the trend of contigs numbers by adding different depth of 454 contigs and Solexa contigs were demonstrated in the Figure A, C and E.

We demonstrated that hybrid assembly pipeline improved the quality of genome draft over any single NGS assembly. We sought to investigate the parameters that influenced the outcomes of hybrid assembly. We used 454, Illumina, and SOLiD data separately as a baseline in secondary assembly, and varied the amount of other NGS data. The results of hybrid assemblies are shown in Figure 7. With the 454 assembly as baseline, the original 352 contigs gradually merged into a smaller set when the coverage depth of either Illumina or SOLiD contigs increased. The reduction in number of contigs was accompanied with increase in N50 size. The outcomes for Illumina were stabilized at 220x coverage depth and at 150x for SOLiD (Figure 7A, B). Further increase in coverage depth resulted in very little changes in both the number of contigs and the N50 size. Thus, 220x coverage depth for Illumina and 150x coverage depth for SOLiD are considered good reference for optimizing experiment design in sequencing microbial genome using the hybrid assembly approach. On the other hand, the SOLiD data are less efficient than Illumina data in creating hybrid assembly, illustrated by the larger optimal number of contigs and smaller maximized N50 size.

Similar results were obtained when using Illumina or SOLiD data as baseline, and varying amount of other NGS data in secondary assembly. With Illumina assembly as baseline, the assembly outcomes for adding 454 data stabilized at 25x coverage depth and at 110x for SOLiD (Figure 7C, D). With SOLiD assembly as baseline, the assembly outcomes for adding 454 data stabilized at 25x coverage depth and at 200x for Illumina (Figure 7E, F).

## Conclusions and discussion

*Enterococcus faecium *is a commensal bacterium inhabiting in the gastrointestinal tract of human, and an important nosocomial pathogen which are often accompanied with multidrug resistance. Some studies determined that most of the *E. faecium *strains from hospital patients belonged to genetic linage termed Complex 17 (CC17) by employing method of multi-locus sequence typing (MLST) [21,22]. The strains from CC17 were often accompanied with pathogenicity island(s), insertion sequence (IS) elements and antibiotic resistance and/or virulence genes [2]. MLST analysis illustrated a genetic difference between the strains from hospital patients and healthy human hosts. Recently, the available multi-strain genome data provided a new insight into these two subpopulations. The core genome analysis had suggested there was a significant difference (3.5-4.2% at the DNA level) between these two subpopulations [23].

The current study attempted to address the difficult issue facing by microbiologists: how to most effectively construct a complete microbial genome using today's state of the art sequencing technologies. In order to accomplish it, we first characterized the sequence data and genome assembly from each NGS platform, then tested various conditions for hybrid assembly with combinations of NGS data, and obtained some optimized parameters for achieving most cost-efficiency assembly. Our results helped form some guidelines to direct genomic work on other analogous microorganisms, and thus had some important practical implications.

In the study, we employed three most popular NGS technologies, Illumina GAIIx, ABI SOLiD4.0, and 454 GS-FLX, each having some unique properties. High coverage depth was obtained with GAIIx and SOLiD, while 454 data were limited to 28-fold because of associated higher cost. Upon applying quality filter, 454 and SOLiD retained over 99% effective sequence reads while GAIIx lost close to one quarter. The larger proportion of low quality sequence reads was repeatedly observed with GAIIx, which warranted some attention to plan compensation for GAIIx experiments. By performing assemblies with each NGS platforms, we obtained some baselines, and upper and lower boundaries for each NGS technology (Figure 2). The distinct results from each NGS were associated with the intrinsic properties of each technology, i.e. sequence read length, base calling error rates, systematic bias, etc. The results indicated that each NGS assembly had a ceiling in continuity that could not be overcome by simply increasing data coverage depth. Previously, the amount and read length of sequencing data were considered as important factors influencing the results of assembled contigs [24,25]. Our work showed that amount of sequence data influenced the assembly outcomes in certain range, and increased coverage depth beyond this range seemed to make very little impact. The longer read length of 454 data generated longer continuous assemblies than Illumina or SOLiD did.

We tested the hybrid assembly approach that integrated data from three next generation sequencing platforms. The N50 size of hybrid assembly was significantly increased over each single NGS results (Table 2). In addition, we also observed marked improvement of N50 in hybrid assembly using two single NGS data. The SOLiD data, although having the shortest read length, helped make much improved assemblies of *E. faecium *genome using the hybrid approach. This was the first such study to combine SOLiD sequence data with other type of NGS data.

The genomic divergence of *E. faecium *strains was analysed by comparing our hybrid assembled genome HEf-3 with 28 partial genome sequences deposited at NCBI. We observed significant alignment differences between these strains and believed the differences were primarily due to *E. faecium *divergent genome with a large number of strain-specific gene contents. Willem and his-coworkers also showed that different *E. faecium *strains contained significantly different gene contents (up to 12%) [1], the acquisition of mobile elements, such as insertion sequence (IS) elements, phage genes, plasmid sequences, antibiotic resistance genes and regulatory genes, mainly contributed to its divergence [2]. In addition, due to the intrinsic biases from sequencing platforms, the alignment differences might reflect some inevitable sequencing errors. Thus, the highly divergent genomes hindered the construction of a novel genome. The de novo assembly approach was urgently needed to be optimized to resolve this issue.

In order to better integrate NGS sequence data, it is important to characterize the biases of each NGS technology. These biases include skewing in coverage depth, bias for particular sequences, i.e. GC contents, k-mer diversity, and unequal tendency in substitution error in different NGS platforms. We observed that the gap regions in each different NGS, in general, had a lower GC content than other regions. Particularly, the gap regions in SOLiD had lowest GC contents of the three. What have not been characterized previously was the detailed differences in bias among different NGS technologies. Although it was showed GC contents had significant influence on coverage depth in all three NGS platforms, the gaps, which are unable to be filled by SOLiD data, was most sensitive to GC contents. For GAIIx data, GC contents affecting k-mers depth might force the high GC repeat sequences to be over displayed. In addition, each NGS showed a different pattern of substitution errors, which were presumably related to the different chemistry of each NGS platform.

Previously, some studies performed hybrid assemblies either using data sets of Sanger, 454, and solexa reads, or two of the three sets. DiGuistini and co-workers combined Sanger PE, 454 SE, and Illumina PE sequence data to perform hybrid assembly [12], in which they used Forge to generate a genome draft with a length 32.5 Mb and N50 size of 32 kb. Though Sanger's longer reads were advantageous in extending an assembly, the generation of 18,424 Sanger reads was associated with much higher cost. Reinhardt et al. assembled their genome using only Solexa and 454 reads. They first assembled short Solexa reads into contigs, before merging these contigs with 454 long reads [11]. However, their secondary assembly using Newbler may result in possible drawbacks for their approach. Besides Newbler was developed and tested mostly on with 454 data, there was a limitation on the length of sequences that can be used as input for secondary assembly. Also the coverage depth of 454 data directly used in secondary assembly could skew the constructed genome as the Illumina contigs from primary assembly could be counted only as one-fold coverage depth.

In order to optimize the hybrid assembly with data from the different NGS technologies, we tested assembly conditions by varying the amount of different NGS data. We observed continuing growth of assembly N50 as the coverage depth of the varying NGS data increased. However, like assembly with single NGS data, there were ceilings for the increase in N50, which were stalled at distinct coverage depths for different NGS data. The progressive results provided us with some basis to optimize future sequence study using the hybrid assembly approach.

Our hybrid assembly approach consisted of primary and secondary assembly steps. Our pipeline could be easily expended by adding primary assembly path for processing data from new platforms, i.e. SMRT, ION PGM, etc. Pacific Biosciences announced that SMRT technology could generate reads with a length more than 1000 bp base pairs [26]. Integration of primary assembly processing SMRT long reads is certainly a focus to extend our pipeline in the future.

### Useful guidelines for hybrid assembly

It is difficult to optimize genome study parameters for hybrid assembly without a thorough understanding of the properties of each NGS technologies. We compared the assemblies from each single NGS data and characterized the systematic biases (coverage, GC content, k-mer diversity, substitution error) of each platform. Based on our investigations, we hope to provide some useful guidelines to help people choose the best strategy, and optimize conditions for hybrid assembly approach. To construct a microbial genome similar in size to that of *E. faecium'*s, sequencing with 454 GS-FLX, which has the best efficiency compared to that of Illumina GAIIx and SOLiD4, is desired. Usually 25-fold coverage depth by 454 GS-FLX is sufficient. Addition of 240-fold Illumina GAIIx or 300-fold SOLiD sequence coverage data will produce a much improved assembly in term of total length, N50, and error correction. If cost allowed, the optimal outcome can be achieved with the combinations of 454 GS-FLX, Illumina GAIIx, and SOLiD4 sequencing data at abovementioned coverage depths. If two types of NGS to be used, the combination of 454 GS-FLX and Illumina GAIIx is preferred over the other two possible combinations.

## List of abbreviations

NGS: Next-Generation Sequencing; SR: single read; PE: pair-end read NCBI: National Center for Biotechnology Information; GR: gap regions; NGR: non-gap regions. HEf-3: Hybrid assembled contigs from three NGS data.

## Competing interests

The authors declare that they have no competing interests.

## Authors' contributions

YJW collected data and performed the experiments. XGX and XL conceived the study, and drafted and revised the manuscript. YY and PBH helped conduct part of the analysis and edit the manuscript. ZFS and YXL advised on experiments and data analysis. All authors read and approved the final manuscript.
